# Genomic Instability of Mutation-Derived Gene Prognostic Signatures for Hepatocellular Carcinoma

**DOI:** 10.3389/fcell.2021.728574

**Published:** 2021-10-05

**Authors:** Ze-Bing Song, Yang Yu, Guo-Pei Zhang, Shao-Qiang Li

**Affiliations:** Department of Liver Surgery, The First Affiliated Hospital of Sun Yat-sen University, Guangzhou, China

**Keywords:** hepatocellular carcinoma, genomic instability, prognostic signature, TCGA, ICGC

## Abstract

Hepatocellular carcinoma (HCC) is one of the major cancer-related deaths worldwide. Genomic instability is correlated with the prognosis of cancers. A biomarker associated with genomic instability might be effective to predict the prognosis of HCC. In the present study, data of HCC patients from The Cancer Genome Atlas (TCGA) and International Cancer Genome Consortium (ICGC) databases were used. A total of 370 HCC patients from the TCGA database were randomly classified into a training set and a test set. A prognostic signature of the training set based on nine overall survival (OS)–related genomic instability–derived genes (SLCO2A1, RPS6KA2, EPHB6, SLC2A5, PDZD4, CST2, MARVELD1, MAGEA6, and SEMA6A) was constructed, which was validated in the test and TCGA and ICGC sets. This prognostic signature showed more accurate prediction for prognosis of HCC compared with tumor grade, pathological stage, and four published signatures. Cox multivariate analysis revealed that the risk score could be an independent prognostic factor of HCC. A nomogram that combines pathological stage and risk score performed well compared with an ideal model. Ultimately, paired differential expression profiles of genes in the prognostic signature were validated at mRNA and protein level using HCC and paratumor tissues obtained from our institute. Taken together, we constructed and validated a genomic instability–derived gene prognostic signature, which can help to predict the OS of HCC and help us to explore the potential therapeutic targets of HCC.

## Introduction

Hepatocellular carcinoma (HCC) is the sixth most prevalent cancer, and its mortality remains the fourth cancer-related deaths worldwide (Bray et al., 2018). The overall survival (OS) rate for HCC remains low because of its late stage at diagnosis and high recurrence after curative treatment (Bruix et al., 2014; Joliat et al., 2017; Zheng et al., 2017).

In the past decades, serum alpha-fetoprotein (AFP) is the traditional biomarker for diagnosis, outcome prediction, and evaluation of response to therapy in HCC. However, the level of AFP is influenced by other factors, such as cancer stage, tumor size, and underlying liver disease. Currently, AFP is lack of sufficient specificity and sensitivity for diagnosis of HCC (Bruix and Sherman, 2011; Kudo, 2015). As a result, the prognosis of HCC patients remains highly heterogeneous. Hence, some effective and sensitive new biomarkers are needed to improve the prognosis of HCC patients.

It is known that genetic mutation plays an important role in the carcinogenesis and progression of cancers. Many previous studies have revealed that genetic mutation of some vital genes has significant relevance to the development and prognosis of HCC. Activating mutations in CTNNB1 are negatively correlated with the prognosis of HCC related to alcoholic cirrhosis (Schulze et al., 2015). While inactivating mutations of TP53 are negatively affected the prognosis of HCC with hepatitis B virus infection (Nishida et al., 1993). Thus, genomic instability may serve as an important prognostic biomarker for cancers (Suzuki et al., 2003; Ottini et al., 2006). Several prognostic signatures based on genomic instability have been constructed for predicting survival of patients with breast cancer (Habermann et al., 2009; Bao et al., 2020) and ovarian cancer (Wang et al., 2017). To date, genomic instability–derived gene biomarkers for HCC have not been explored. In the present study, we construct a genomic instability–derived genes (GIGs) prognostic signature (GIGSig) to predict the OS of patients with HCC and explore the potential therapeutic targets of HCC based on The Cancer Genome Atlas (TCGA) and International Cancer Genome Consortium (ICGC) databases.

In addition, UBQLN4 is closely correlated with genomic instability and overexpressed in various malignancies (Jachimowicz et al., 2019). UBQLN4 may act as a biomarker for genomic instability. Therefore, we also analyzed the expression profile of UBQLN4 between genomic-unstable (GU)–like group and genomic-stable (GS)–like group with the aim to evaluate the genomic instability in different groups of patients with HCC.

## Materials and Methods

### Data Source

The transcriptomic profiling of RNA sequencing data, simple nucleotide variation data, and clinical data of HCC patients were collected from the TCGA database. The patients were randomly categorized into a training (*n* = 186) and test (*n* = 184) set. The demographic characteristics of the training, test and TCGA sets were shown in Table 1. The clinicopathological variables were not significantly different between the training and test sets. To externally validate the predictive value of the prognostic signature, the simple nucleotide variation data and the LIRI-JP data set from the ICGC database were used.

**TABLE 1:** Clinicopathological parameters of HCC patients in the training, test, and TCGA data sets.

**Covariates**	**Type**	**Total**	**Training**	**Test**	***P*-value**
Age	≤65 years	232 (63%)	111 (60%)	121 (66%)	0.2703
	>65 years	138 (37%)	75 (40%)	63 (34%)	
	Unknown	0 (0%)	0 (0%)	0 (0%)	
Gender	Female	121 (33%)	63 (34%)	58 (32%)	0.7108
	Male	249 (67%)	123 (66%)	126 (68%)	
Grade	G1–2	232 (63%)	112 (60%)	120 (65%)	0.4063
	G3–4	133 (36%)	71 (38%)	62 (34%)	
	Unknown	5 (1%)	3 (2%)	2 (1%)	
Stage	I–II	256 (69%)	122 (66%)	134 (73%)	0.2433
	III–IV	90 (24%)	50 (27%)	40 (22%)	
	Unknown	24 (6%)	14 (8%)	10 (5%)	
T	T1–2	274 (74%)	132 (71%)	142 (77%)	0.282
	T3–4	94 (25%)	52 (28%)	42 (23%)	
	Unknown	2 (1%)	2 (1%)	0 (0%)	
M	M0	266 (72%)	132 (71%)	134 (73%)	0.6144
	M1	4 (1%)	3 (2%)	1 (0.5%)	
	Unknown	100 (27%)	51 (27%)	49 (26.5%)	
N	N0	252 (68%)	125 (67%)	127 (69%)	0.6143
	N1	4 (1%)	3 (2%)	1 (0.5%)	
	Unknown	114 (31%)	58 (31%)	56 (30.5%)	

### Identification of Genomic Instability–Derived Genes in the Cancer Genome Atlas and International Cancer Genome Consortium Data Sets

The mutations that cause amino acid changes are defined as gene mutations. The cumulative numbers of gene mutations for each sample in the TCGA and ICGC sets were calculated by the Perl software. Subsequently, samples were ranked and classified into GU-like group (the top 25% of patients) and GS-like group (the last 25% of patients) according to the number of gene mutations. Then, differentially expressed gene analyses between GS-like and GU-like group were performed using the Limma R package; | log2 fold change| > 1 and false discovery rate <0.05 were set as the cutoff values. Genes differentially expressed in both the TCGA and ICGC data sets were defined as GIGs. The Gene Ontology (GO) and Kyoto Encyclopedia of Genes and Genomes (KEGG) function analyses were performed to explore the potential molecular function of these GIGs. Based on GIGs, hierarchical cluster analyses of TCGA and ICGC sets were performed using Euclidean distances and Ward’s linkage method.

### Development and Validation of the Genomic Instability–Derived Gene Prognostic Signature

We then developed a GIGSig in the training set and validated in the test and TCGA and ICGC sets. The OS was selected as the primary efficacy end point in this study. Cox univariate analysis was performed in the training set to identify the OS-related GIGs; *P* < 0.05 was selected as the cutoff value. Least absolute shrinkage and selection operator (LASSO) analysis was performed to avoid overfitting the prognostic signature. Cox multivariate analysis was performed to construct a GIGSig. Kaplan–Meier curves were plotted to compare the OS of high- and low-risk groups. The receiver operating characteristic (ROC) curve was plotted, and the area under the ROC (AUC) was calculated to evaluate the predictive efficacy of this signature. The clinicopathological features and risk score were analyzed by univariate and multivariate Cox analyses to explore the risk factors that affected the survival of HCC patients. Subgroup analysis and the correlation analysis between the risk score and clinicopathological characteristics were performed.

### Development and Validation of a Nomogram for Predicting OS of Hepatocellular Carcinoma

A nomogram that integrated the independent prognostic factors selected from multivariate regression analysis was developed to predict the OS of HCC patients at 1, 3, and 5 years. The prediction accuracy of this nomogram was validated by comparing the observed actual probability with the calibration curve.

### Tissue Samples

Thirty HCC tissues and paired adjacent non-cancerous liver tissues were collected from patients who underwent liver resection in the First Affiliated Hospital of Sun Yat-sen University from June 2019 to December 2019. The samples were snap-frozen in liquid nitrogen and stored at −80°C before RNA and protein extraction. This study was approved by the Ethics Committee of the First Affiliated Hospital of Sun Yat-sen University [Approval number (2021)158].

### Quantitative Real-Time Reverse Transcription Polymerase Chain Reaction

Total RNA was isolated from the tissues using TRIZOL reagent (Invitrogen, CA, United States) according to the manufacturer’s instructions. Complement DNAs (cDNAs) were reverse transcribed using SuperScript First-Stand Synthesis system (Invitrogen, Carlsbad, CA, United States). The synthesized cDNAs were used for real-time polymerase chain reaction (PCR) with reverse transcriptase–PCR System (Roche, LightCycler480 II, Switzerland), and the conditions were performed according to the manufacturer’s instructions. The primers of GAPDH were used as internal loading control. All primers are shown in Table 2. Samples were normalized to internal housekeeping genes. All the values were standardized with 2^–Δ^ ^Δ^ ^CT^ method.

**TABLE 2:** The primers of genes.

**Genes**	**Forward primers**	**Reverse primers**
SLCO2A1	5′-ACTCTTCGTCCTGGTGGTCCTG-3′	5′-CTGCTGAGGTGCCATACTGCTTC-3′
SLC2A5	5′-GGATGAGGTGGCTTTGAGTGATGG-3′	5′-ACTGGTGGCTGTCATTGCTATGC-3′
SEMA6A	5′-GCAGGACATAGAGCGTGGCAATAC-3′	5′-CTGGGCAAGAGGGAACTGGAATG-3′
PDZD4	5′-CTGAAAGCCCGTGCCCTGAAG-3′	5′-TTCCGCTCCTCCTTGCTCCAG-3′
MAGEA6	5′-GCATGAGTGGGCTTTGAGAGAGG-3′	5′-CGTCACAGGAGGCAGTGGAAAC-3′
EPHB6	5′-CGACAGCCCTGACAGCGTTTC-3′	5′-GCAGAGGAAGAAGAGGAGGAGGAG-3′
CST2	5′-CCCAGGAGGAGGACAGGATAATCG-3′	5′-ACTCGCTGATGACAAAGTGAAGGG-3′
RPS6KA2	5′-GAGGAGGATGTCAAGTTCTACC-3′	5′-CTCAGGCTTCAGATCTCTGTAG-3′
MARVELD1	5′-CCCAGGATGAGCGACGAGTTTG-3′	5′-CAAGACAACCGAGCACAGAGAGAC-3′
GAPDH	5′-ACAACTTTGGTATCGTGGAAGG-3′	5′-GCCATCACGCCACAGTTTC-3′

### Western Blot

We extracted proteins by using RIPA lysis buffer with 1% proteinase inhibitor and quantified by a BCA kit (Thermo, United States). Equal amounts of proteins (20 μg) were separated by 7.5, 10, or 12.5% sodium dodecyl sulfate–polyacrylamide gel electrophoresis based on the molecular weight of the proteins and transferred onto polyvinylidene fluoride membranes (Millipore, United States). After blocking for 2 h with 5% skim milk powder at room temperature, membranes were incubated with primary antibodies at 4°C overnight. The primary antibodies are shown in Supplementary Table 1. The membranes were then incubated with horseradish peroxidase (HRP)–conjugated goat anti-rabbit secondary antibody (1:5,000, Abcam, United Kingdom) for 11/2 h and visualized using the Immobilon Western Chemiluminescent HRP Substrate (Millipore, United States).

### Statistical Analysis

All the statistical analysis was performed by R software (version 3.6.1) and Perl software (version 5.30). *P* < 0.05 was set as the cutoff value of significance.

## Results

### Identification of Genomic Instability–Derived Genes and Development of a Genomic Instability–Derived Gene Prognostic Signature

**FIGURE 1:**
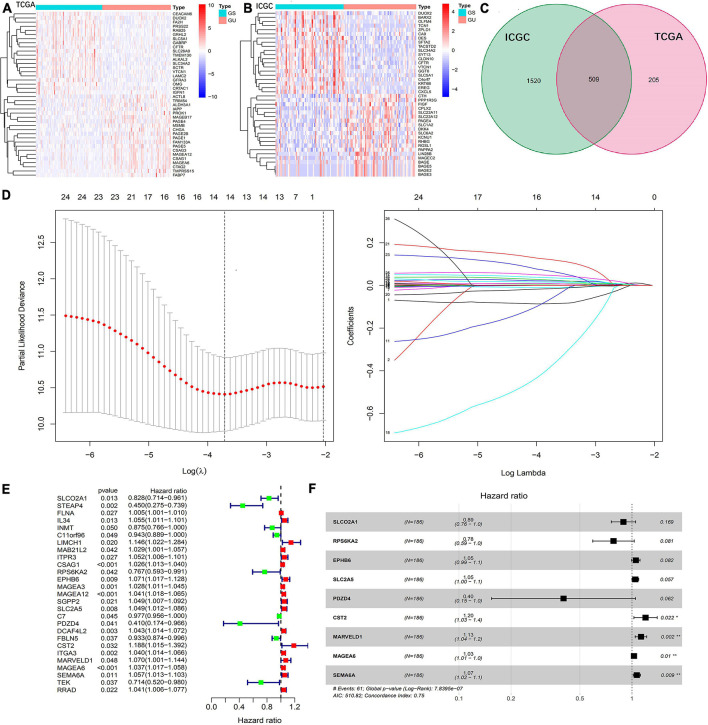
Identification of GIGs and construction of a GIGSig. **(A,B)** Heatmaps of the top 20 differentially expressed genes between GS-like and GU-like group of the TCGA and ICGC profiles, respectively. **(C)** Intersect of GIGs of the TCGA and ICGC sets. **(D)** The results of LASSO analysis of OS-related GIGs. **(E)** Univariate Cox regression analysis of GIGs to identify 27 OS-related GIGs. **(F)** Multivariable Cox regression analysis to construct a nine-gene-based prognostic signature for HCC.

A total of 363 samples of TCGA and 270 samples of ICGC data set were identified as gene mutation samples. Gene differential expression analysis identified 656 down-regulated and 58 up-regulated genes in the TCGA set (Figure 1A) and 1,853 down-regulated and 176 up-regulated genes in the ICGC set (Figure 1B). Subsequently, 509 genes were identified to be differentially expressed in both the TCGA and ICGC sets, which were defined as GIGs in the present study (Figure 1C).

Gene Ontology analysis of these GIGs revealed that “extracellular matrix organization,” “collagen-containing extracellular matrix,” and “extracellular matrix structural constituent” were the most frequently involved biological terms under the biological processes, cellular components, and molecular functions, respectively (Supplementary Figure 1A). In the KEGG analysis, the main pathways related to these GIGs were the PI3K-Akt pathway, the Wnt pathway, and the MAPK pathway (Supplementary Figure 1B).

Based on the expression levels of the 509 GIGs, unsupervised hierarchical clustering analysis was performed on 374 samples of the TCGA set (Supplementary Figure 1C) and 237 samples of the ICGC set (Supplementary Figure 1D). The samples were categorized as the GS-like and GU-like groups. Somatic mutations counts were significantly higher in the GU-like group than that in the GS-like group in both TCGA (Supplementary Figure 1E) and ICGC sets (Supplementary Figure 1F). UBQLN4 is one of the key genes driving biological process of genomic instability. Therefore, the expression levels of UBQLN4 between the GS-like and GU-like groups were measured. Higher expression of UBQLN4 was identified in the GU-like group when compared to the GS-like group in both the TCGA (Supplementary Figure 1G) and ICGC sets (Supplementary Figure 1H).

Among them, 27 of 509 GIGs identified by the Cox univariate analysis were correlated with the OS in the training set (Figure 1E), and 14 of 27 OS-related GIGs were suitable to establish a prognostic signature based on the LASSO analysis (Figure 1D). Finally, 9 of 14 GIGs were selected by the Cox multivariate analysis to construct a prognostic signature (Figure 1F).

The prognostic signature was presented as a risk score that was calculated by multiplying the gene expression levels and Cox regression coefficients. The formula was as follows: risk score = (expression level of SLCO2A1 × −0.1137) + (expression level of RPS6KA2 × −0.2461) + (expression level of EPHB6 × 0.0503) + (expression level of SLC2A5 × 0.0441) + (expression level of PDZD4 × −0.9111) + (expression level of CST2 × 0.1804) + (expression level of MARVELD1 × 0.1193) + (expression level of MAGEA6 × 0.0266) + (expression level of SEMA6A × 0.0645).

### Evaluation and Validation of the Genomic Instability–Derived Gene Prognostic Signature

**FIGURE 2:**
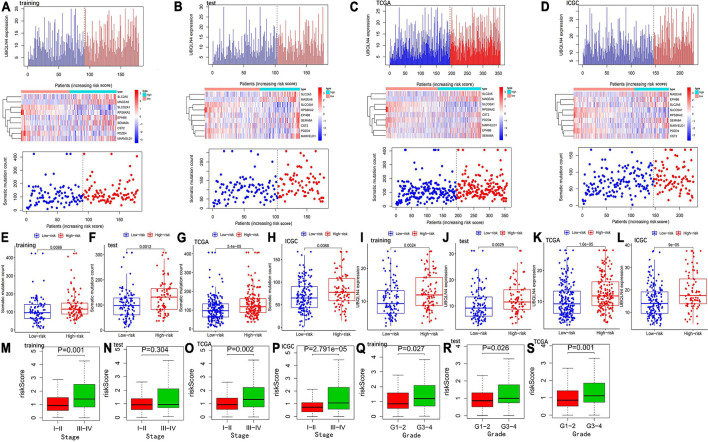
Evaluation of the GIGSig in the training, test, TCGA, and ICGC sets. The expression patterns of GIGs in GIGSig and the distribution of somatic mutation count distribution and UBQLN4 expression for patients in high- and low-risk groups in the training set **(A)**, test set **(B)**, TCGA set **(C)**, and ICGC set **(D)**. The distribution of somatic mutation and UBQLN4 expression in patients of high- and low-risk groups in the training set **(E,I)**, test set **(F,J)**, TCGA set **(G,K)**, and ICGC set **(H,L)**. Box plot of correlation between the risk score and clinical stages in the training set **(M)**, test set **(N)**, TCGA set **(O)**, and ICGC set **(P)**. Box plot of correlation between the risk score and tumor grade in the training set **(Q)**, test set **(R)**, and TCGA set **(S)**. Horizontal lines: median values.

Hepatocellular carcinoma patients of the training, test, TCGA, and ICGC sets were categorized into the high- and low-risk groups by the median value of the risk score in the training set. The heatmap analysis indicated that six GIGs were positively related to the risk score, and the other three GIGs were negatively associated with the risk score. Somatic mutation count and UBQLN4 expression of patients were positively related to the risk score (Figure 2). Furthermore, patients in the high-risk group have a higher somatic mutation count and higher UBQLN4 expression compared with those in the low-risk group in the training, test, TCGA, and ICGC sets (Figure 2). Clinical correlation analysis revealed that the risk score was related to pathological stage and tumor grade in the training and TCGA and ICGC sets (Figure 2).

**FIGURE 3:**
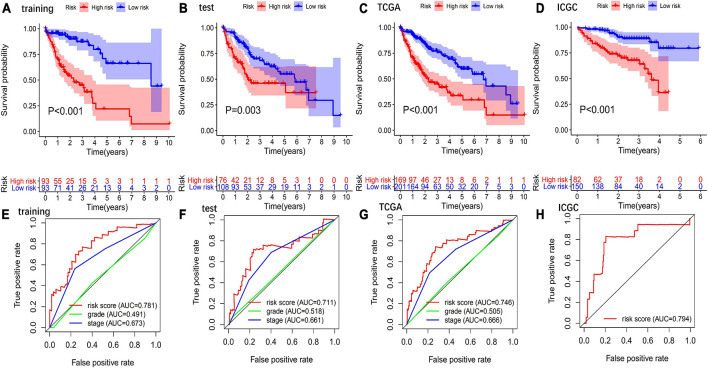
Evaluation and validation of the predictive values of GIGSig. Kaplan–Meier analysis of OS of patients in high- and low-risk groups predicted by the GIGSig in the training set **(A)**, test set **(B)**, TCGA set **(C)**, and ICGC set **(D)**. ROC curve analysis of the GIGSig of the training set **(E)**, test set **(F)**, TCGA set **(G)**, and ICGC set **(H)**.

Patients in the high-risk group had a significantly poorer OS than those in the low-risk group in the training (Figure 3A), test (Figure 3B), TCGA (Figure 3C), and ICGC sets (Figure 3D).

The AUCs of the GIGSig in the training (Figure 3E), test (Figure 3F), TCGA (Figure 3G), and ICGC sets (Figure 3H) were 0.781, 0.711, 0.746, and 0.794, respectively. Furthermore, the AUCs of the GIGSig were markedly higher than the AUCs of pathological stage and tumor grade, respectively.

Cox univariate and multivariate analyses revealed that the risk score and pathological stage were independent prognostic factors for patients in the training, test, ICGC, and TCGA sets (Table 3).

**TABLE 3:** Univariate and multivariate cox regression analysis of the GIGSig and clinicopathologic factors in different patient sets.

**Variables**		**Univariate analysis**		**Multivariate analysis**	
		**Hazard ratio (95% CI)**	***P*-value**	**Hazard ratio (95% CI)**	***P*-value**
**Training set (*n* = 186)**					
Age		1.006 (0.983–1.029)	0.587	NA	NA
Gender	Male/female	1.322 (0.751–2.329)	0.333	NA	NA
Tumor grade	G1/G2/G3/G4	1.315 (0.898–1.926)	0.160	NA	NA
Pathologic stage	I/II/III/IV	1.706 (1.270–2.291)	<0.001	1.519 (1.112–2.075)	0.008
Risk score	High/low	1.219 (1.145–1.298)	<0.001	1.187 (1.112–1.267)	<0.001
**Test set (*n* = 184)**					
Age		1.013 (0.994–1.033)	0.187	NA	NA
Gender	Male/female	0.470 (0.279–0.793)	0.004	0.496 (0.293–0.837)	0.008
Tumor grade	G1/G2/G3/G4	1.013 (0.722–1.422)	0.94	NA	NA
Pathologic stage	I/II/III/IV	1.737 (1.284–2.352)	<0.001	1.693 (1.251–2.292)	<0.001
Risk score	High/low	1.135 (1.039–1.294)	<0.001	1.097 (1.015–1.171)	0.006
**TCGA set (*n* = 370)**					
Age		1.01 (0.996–1.025)	0.174	NA	NA
Gender	Male/female	0.776 (0.531–1.132)	0.188	NA	NA
Tumor grade	G1/G2/G3/G4	1.133 (0.881–1.457)	0.329	NA	NA
Pathologic stage	I/II/III/IV	1.680 (1.369–2.061)	<0.001	1.672 (1.363–2.052)	<0.001
Risk score	High/low	1.075 (1.04–1.111)	<0.001	1.079 (1.041–1.120)	<0.001
**ICGC set (*n* = 232)**					
Age		1.003 (0.973–1.034)	0.841	NA	NA
Gender	Male/female	0.516 (0.277–0.961)	0.037	0.40 (0.212–0.756)	0.004
Pathologic stage	I/II/III/IV	2.138 (1.481–3.087)	<0.001	2.316 (1.602–3.348)	<0.001
Risk score	High/low	1.219 (1.096–1.356)	0.005	1.118 (1.056–1.268)	<0.001

### Performance Comparison of the Genomic Instability–Derived Gene Prognostic Signature With Previous Gene Signatures for OS Prediction in HCC Patients

**FIGURE 4:**
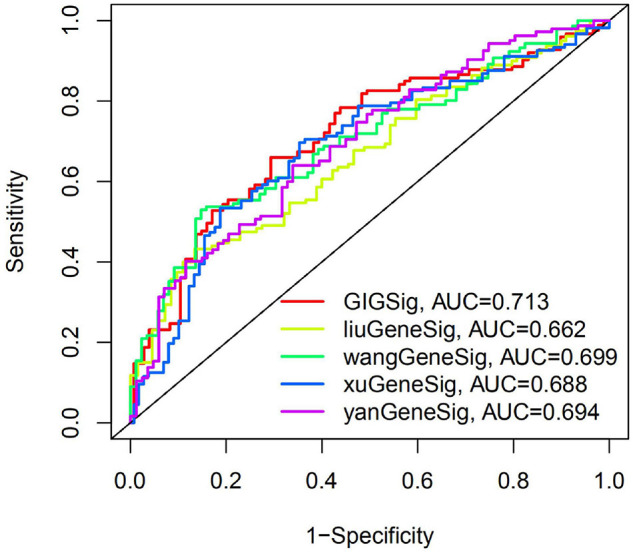
The AUC at 3 years of OS for the GIGSig and the four previously reported gene-based signatures.

To further evaluate the predictive efficacy of GIGSig on OS, we compared the AUC of OS at 3 years of GIGSig with other four published gene signatures using the same TCGA data set. These four signatures included a six-gene-based signature by Liu et al. (2019), a nine-immune-gene-based signature by Wang et al. (2020), a 6-immune-gene-based signature by Xu et al. (2021), and a 4-gene-based signature by Yan et al. (2019). The AUC of OS at the 3-year of GIGSig was 0.713, which was higher than Liu’s (0.622), Wang’s (0.699), Xu’s (0.688), and Yan’s signature (0.694) (Figure 4). These results suggested that the predictive performance of the present GIGSig is more accurate than the above four published gene signatures.

### Subgroup Analysis

Subgroup analysis of the training, test, TCGA, and ICGC sets were performed based on age (>65 and ≤65 years), gender (female and male), tumor grade (grades 1–2 and grades 3–4), pathological stage (stages I–II and III–IV), tumor stages (T1–2 and T3–4), distant metastasis status (M0), and lymph node metastasis status (N0). In each subgroup of the training set (Supplementary Figure 2), TCGA set (Supplementary Figure 4), and ICGC set (Supplementary Figure 2), subgroup analysis showed that GIGSig performed well in predicting OS of HCC patients. While in subgroup analysis of the test set, except for patients in subgroups of age >65 years and G3–4, patients of other subgroups in the high-risk group have poorer OS than those in the low-risk group (Supplementary Figure 3).

### Construction of a Nomogram for Predicting OS of Hepatocellular Carcinoma

**FIGURE 5:**
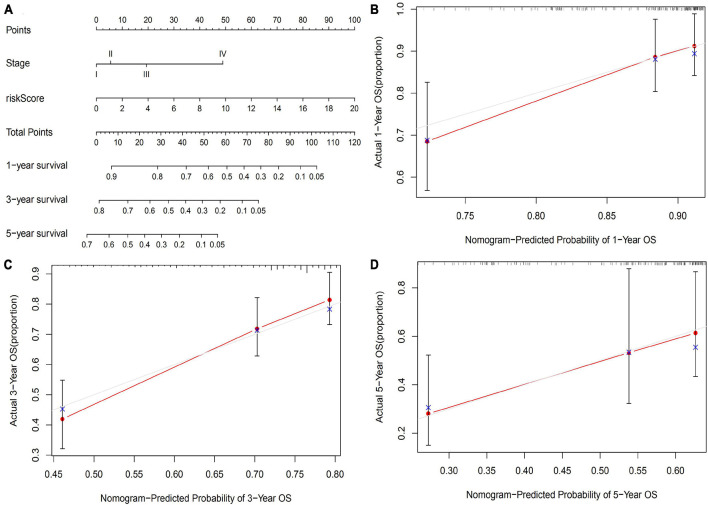
Construction of an OS predicted nomogram. **(A)** A nomogram integrates the risk score of GIGSig with pathological stage to predict the OS at 1, 3, and 5 years of HCC. Calibration curves of OS at 1 **(B)**, 3 **(C)**, and 5 years **(D)**.

As shown in Figure 5, we developed a 1-, 3-, and 5-year OS predictive nomogram by integrating the risk score and pathological stage. Calibration plots showed that our nomogram performed well in OS prediction.

### Paired Differential Expression Analysis of Genes Included in Genomic Instability–Derived Gene Prognostic Signature

**FIGURE 6:**
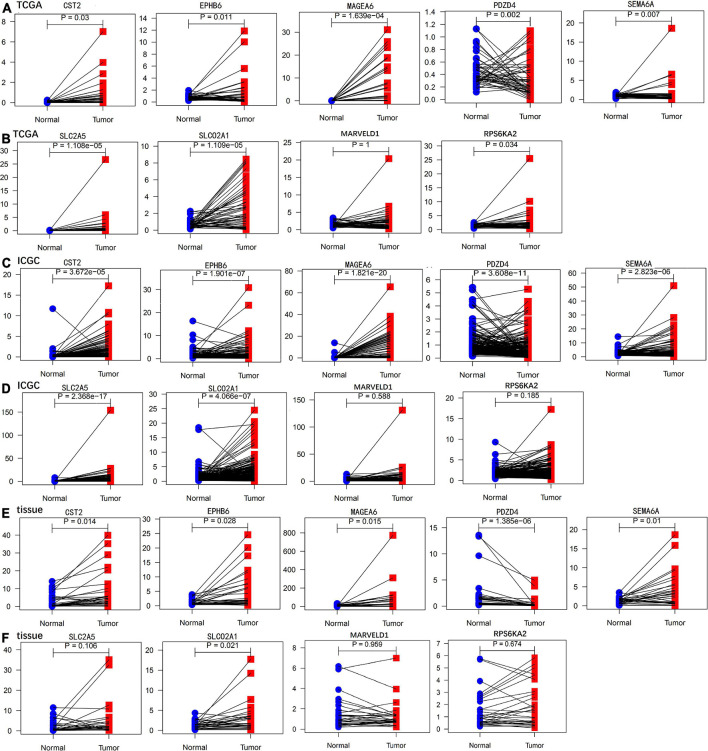
Paired differential expression analysis of genes in the GIGSig. **(A,B)** Paired differential expression profiles of genes in the TCGA samples. **(C,D)** Paired differential expression profiles of genes in the ICGC samples. **(E,F)** Paired differential mRNA expression of genes in the HCC tissues from our institute.

We performed paired differential expression analysis to explore the aberrant expression of the nine genes recruited in GIGSig between HCC samples and non-tumor liver samples in the TCGA and ICGC sets. Fifty paired tissues in the TCGA set and 198 paired tissues in the ICGC set were included. The results showed that the mRNA expressions of seven genes in both TCGA and ICGC sets were significantly different between HCC samples and non-tumor samples, including six genes (CST2, EPHB6, MAGEA6, SEMA6A, SLC2A5, and SLCO2A1), and were highly expressed in HCC samples and one gene (PDZD4) expressed low in HCC samples (Figure 6).

### Validation of the mRNA and Protein Expression Profile of Genomic Instability–Derived Gene Prognostic Signature in Clinical Tissues

**FIGURE 7:**
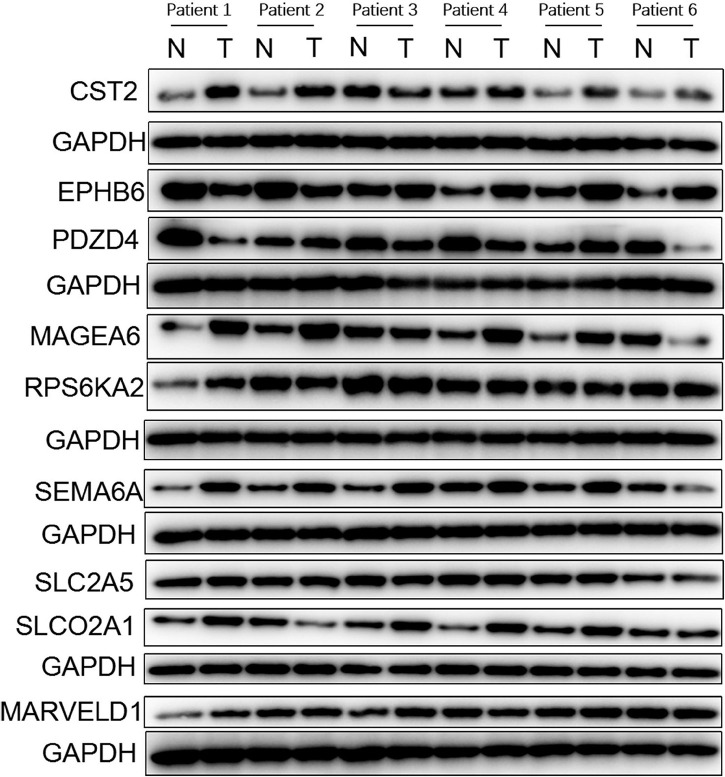
Protein expression profiles of genes in the GIGSig analyzed based on normal and tumor tissues from six patients diagnosed with HCC in the institute of The First Affiliated Hospital of Sun Yat-sen University between the years June 2019 to December 2019. N, normal tissue; T, tumor tissue.

We further validated the differential mRNA expression level of the above nine genes in 30 pairs of HCC tissues and adjacent non-cancerous liver tissues collected from our hospital. The results showed that the mRNA levels of five genes (CST2, EPHB6, MAGEA6, SEMA6A, and SLCO2A1) were significantly elevated in the HCC tissues, whereas PDZD4 was down-regulated in the HCC tissues when compared with those in the paratumor tissues (Figure 6). These results were consistent with the results of TCGA and ICGC profile. In addition, the protein levels of the above nine genes in six pairs of HCC tissues and paratumor tissues were detected by using Western blot. As shown in Figure 7, the protein level of CST2 was significantly elevated in the HCC tissues of patients 1, 2, 4, 5, and 6, whereas it was decreased in patient 3. The protein level of EPHB6 was significantly elevated in the HCC tissues of patients 3, 4, 5, and 6 and decreased in patients 1 and 2. The protein level of MAGEA6 was significantly elevated in the HCC tissues of patients 1, 2, 4, and 5 and decreased in patients 3 and 6. The protein level of SEMA6A significantly elevated in the HCC tissues of patients 1, 2, 3, 4, and 5 and decreased in patient 6. The protein level of SLCO2A1 was significantly elevated in the HCC tissues of patients 1, 3, 4, and 5 and decreased in patient 2, and there was no significant difference in patient 6. The protein level of PDZD4 was significantly decreased in HCC tissues of patients 1, 3, 4, and 6 and increased in patients 2 and 5. The protein levels of RPS6KA2, MARVELD1, and SLC2A5 had no significant difference between the HCC tissues and paratumor tissues.

## Discussion

Genomic instability plays an important role in the development, progression, and recurrence of various cancers (Bartkova et al., 2005; Gorgoulis et al., 2005; Kronenwett et al., 2006; Mettu et al., 2010; Negrini et al., 2010). Therefore, genomic instability could be a promising biomarker to predict the oncological outcome of patients with cancers. In this present study, we constructed a novel survival predictive model for HCC patients by using nine genes derived from genome instability.

With a series of bioinformatics analysis by using the HCC database in TCGA, a novel OS predictive model, namely, GIGSig, based on the nine genes derived from genome instability was generated. Clustering analysis revealed that patients in the GU-like group have higher somatic mutation count and higher UBQLN4 expression than those in the GS-like group. Somatic mutation count and UBQLN4 expression level are positively correlated with genome instability process (Jachimowicz et al., 2019). Our results of clustering analysis revealed that these genes are associated with genome instability. In addition, GO and KEGG functional analysis revealed that the biological functions of these genes are mainly correlated with the development and progression of cancers. These results reveal that genes derived from genome instability may play a vital role in HCC prognosis and could be used to construct a prognostic signature.

According to GIGSig score, HCC patients could be divided into two distinct groups: the high-risk group and the low-risk group. Patients in the high-risk group had poorer OS, higher somatic mutation count, and higher UBQLN4 expression than those in the low-risk group. Subgroup analysis indicated that GIGSig can discriminate patients with poor OS, even though they were with early-stage tumor classified by conventional TNM staging. The AUC of GIGSig is the highest compared with the pathological stage, tumor grade, and the four published gene-based prognostic signatures. These results collectively supported that our GIGSig is an effective prognostic signature to predict the OS of HCC patients.

In addition, the present signature was validated externally and internally, whereas the published gene-based signatures were validated only externally. We also performed subgroup analysis to further validate the predictive value of the signature for HCC patients in different statuses classified by clinical features. The results showed that the signature performed well. This result indicated that the gene signature we developed is more clinically meaningful compared with published articles.

To integrate GIGSig with other OS predictive factors, a nomogram model was proposed. Because the GIGSig score and pathological stage were independent OS predictors for HCC, we constructed a nomogram by integrating the GIGSig score and pathological stage to predict the OS of HCC. The GIGSig-based nomogram performed well in OS prediction, which was proved by the calibration curve. Therefore, our GIGSig-based nomogram may be a potential accurate predictive model for predicting the OS of HCC.

Nine genes in GIGSig were SLCO2A1, RPS6KA2, EPHB6, SLC2A5, PDZD4, CST2, MARVELD1, MAGEA6, and SEMA6A. These genes and their biological functions have been studied in some tumors. SLCO2A1 has been reported to promote the development of colon cancer by PGE2 uptake into the endothelial cells (Nakanishi et al., 2017). Metabolic abnormalities of EPHB6 promote cancer development and progression, which was proven in invasive melanoma (Hafner et al., 2003) and prostate, gastric, and ovarian cancers (Eph Nomenclature Committee, 1997). SLC2A5 is an oncogene and up-regulated in some cancers, that is, lung cancer (Weng et al., 2018) and acute myeloid leukemia (Zhao et al., 2018). Previous studies showed that CST2 was expressed higher in breast cancer tissues compared with normal mammary tissues (Egland et al., 2003). High CST2 expression led to a poor prognosis in gastric cancer patients (Zhang et al., 2020). MAGEA6 was identified as an oncogene and can promote tumor progression *via* activating the AMPK signaling pathway (Pineda et al., 2015). PDZD4 is up-regulated in synovial sarcomas, and high expression of PDZD4 promotes the proliferation capacity of synovial sarcomas cells (Nagayama et al., 2004).

SEMA6A is down-regulated in lung cancer cells. High expression of SEMA6A suppressed cancer cell migration (Chen et al., 2019). Although in the work of Yu et al. (2012), MARVELD1 was down-regulated in HCC tissues, and high expression of MARVELD1 suppressed proliferation and enhanced chemosensitivity of HCC cells, our results showed that there was no significant differential expression of MARVELD1 in both TCGA and ICGC paired tumor and normal samples. Genetic variation of RPS6KA2 was related to the carcinogenesis of colorectal cancer (Slattery et al., 2011). RPS6KA2 was also identified as a cancer suppressor gene in epithelial ovarian cancer (Bignone et al., 2007).

We validated the expression profiles of the nine genes in the GIGSig at mRNA and protein levels using paired HCC and paratumor tissues from our department. The results showed that the mRNA expressions of six genes (CST2, EPHB6, MAGEA6, PDZD4, SEMA6A, and SLCO2A1) were differentially expressed between the HCC tissues and paratumor liver tissues from the TCGA, the ICGC, and our clinical samples. In addition, we also validated the protein expression level of these nine GIGs by using Western blot. The protein expression patterns were consistent with the mRNA. Notably, the expression profiles of these nine genes in HCC were somewhat different from their expression patterns in other cancers. This may be due to the different disease entities and the small sample size of our validated cohort.

Although preliminary *in vitro* experiments have verified the differential expression of these GIGs in HCC and paratumor liver tissues, the small sample size used for protein validations is the limitation of our research. Therefore, further *in vivo* and *in vitro* experiments are needed to explore the molecular mechanism of these GIGs in HCC.

To conclude, we constructed and validated a GIGSig that can help in predicting the OS of patients with HCC and may help us to explore the potential therapeutic targets of HCC.

## Data Availability Statement

The datasets presented in this study can be found in online repositories. The names of the repository/repositories and accession number(s) can be found below: TCGA data portal (https://portal.gdc.cancer.gov/), ICGC data base (https://dcc.icgc.org/).

## Ethics Statement

This study was approved by the Ethics Committee of the First Affiliated Hospital of Sun Yat-sen University. Approval number (2021)158.

## Author Contributions

S-QL: conceptualization and manuscript revision. Z-BS, G-PZ, and YY: data curation. Z-BS: tissue validation, data analysis and figure plot, and manuscript writing. All authors approved the final version of the manuscript.

## Conflict of Interest

The authors declare that the research was conducted in the absence of any commercial or financial relationships that could be construed as a potential conflict of interest.

## Publisher’s Note

All claims expressed in this article are solely those of the authors and do not necessarily represent those of their affiliated organizations, or those of the publisher, the editors and the reviewers. Any product that may be evaluated in this article, or claim that may be made by its manufacturer, is not guaranteed or endorsed by the publisher.

## Supplementary Material

Supplementary Figure 1 Functional exploration of GIGs and unsupervised clustering of HCC cancer patients in both the TCGA and ICGC profiles based on the expression pattern of GIGs. **(A)** Bar plot and bubble plot of GO analysis for GIGs. **(B)** Bar plot and bubble plot of KEGG analysis for GIGs. **(C,D)** Heatmaps of genes clustered in GS-like and GU-like group of the TCGA and ICGC profiles. **(E,F)** Box plots of somatic mutations in the GU-like group and GS-like group of the TCGA and ICGC profiles. **(G,H)** Box plots of UBQLN4 expression level in the GU-like group and GS-like group of the TCGA and ICGC profile.

Supplementary Figure 2 Subgroup analysis of the training set **(A,B)** and ICGC set **(C)**.

Supplementary Figure 3 Subgroup analysis of the test set.

Supplementary Figure 4 Subgroup analysis the TCGA set.
