# Supplementary figures and images for: Genomic Instability of Mutation-Derived Gene Prognostic Signatures for Hepatocellular Carcinoma
Source: Front Cell Dev Biol. 2021 Oct 5;9:728574. doi: 10.3389/fcell.2021.728574 (PMC8523793; doi:10.3389/fcell.2021.728574)

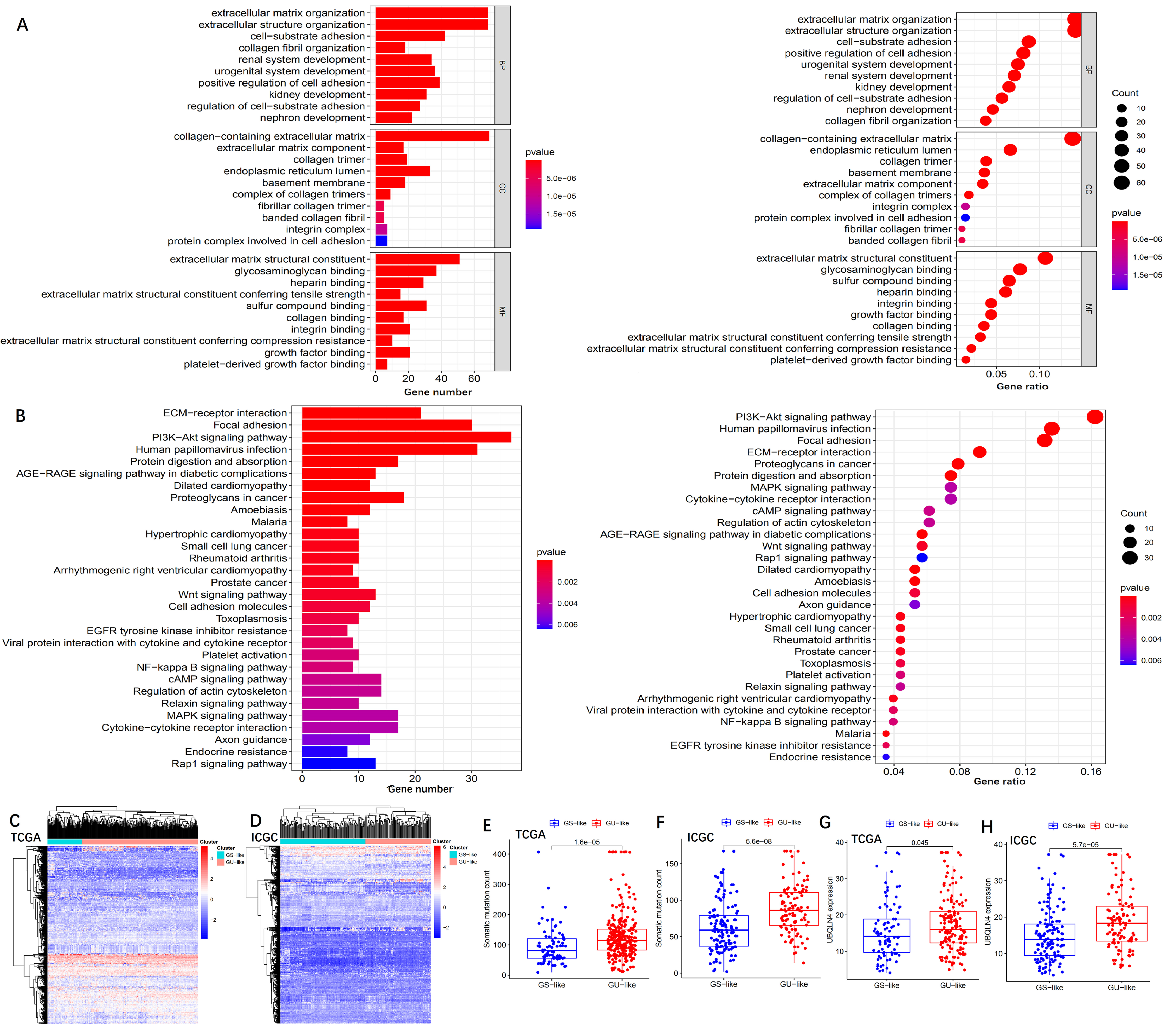

Supplement: Supplementary Figure 1 — Functional exploration of GIGs and unsupervised clustering of HCC cancer patients in both the TCGA and ICGC profiles based on the expression pattern of GIGs. (A) Bar plot and bubble plot of GO analysis for GIGs. (B) Bar plot and bubble plot of KEGG analysis for GIGs. (C,D) Heatmaps of genes clustered in GS-like and GU-like group of the TCGA and ICGC profiles. (E,F) Box plots of somatic mutations in the GU-like group and GS-like group of the TCGA and ICGC profiles. (G,H) Box plots of UBQLN4 expression level in the GU-like group and GS-like group of the TCGA and ICGC profile. [file Image_1.TIF]

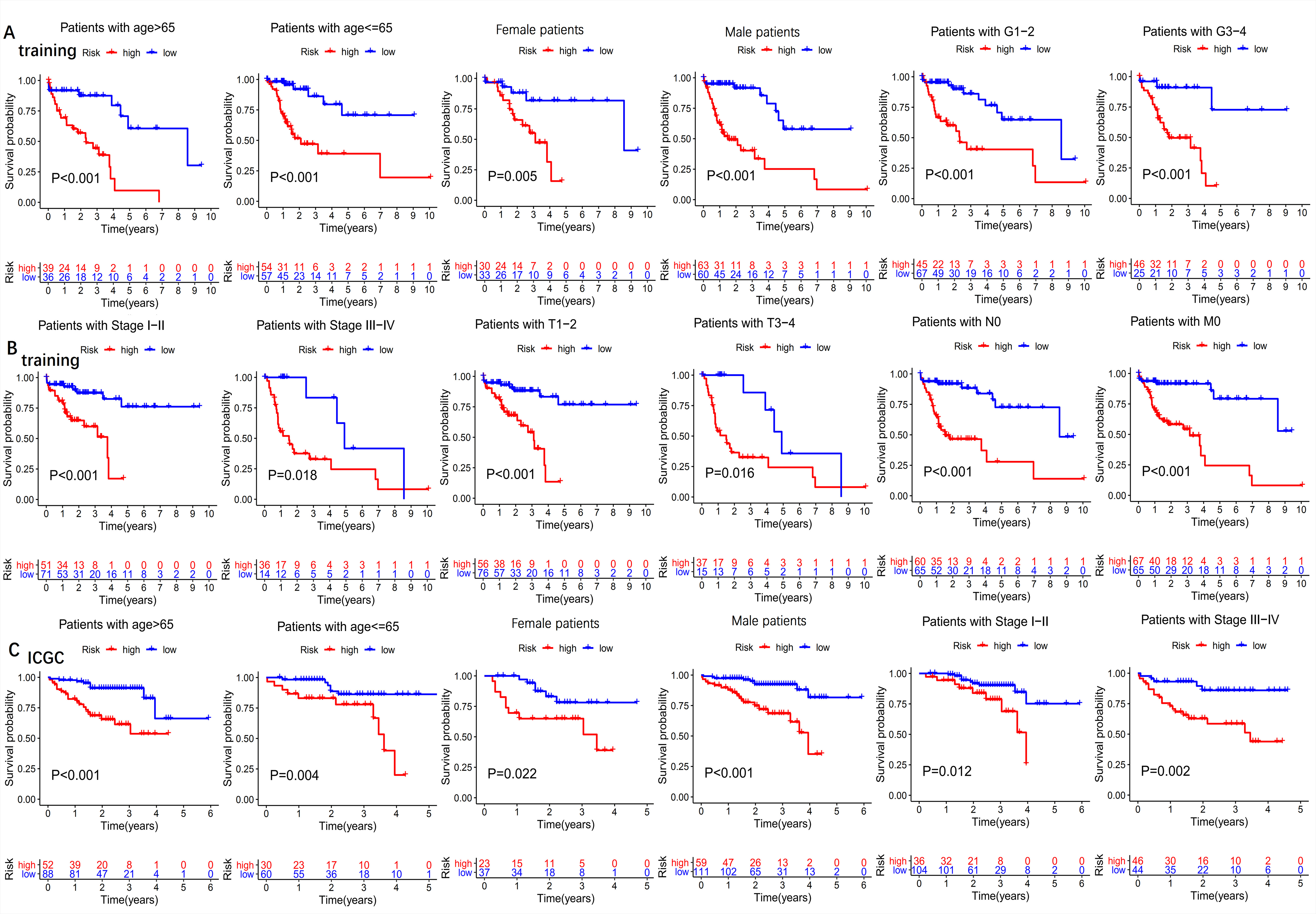

Supplement: Supplementary Figure 2 — Subgroup analysis of the training set (A,B) and ICGC set (C). [file Image_2.TIF]

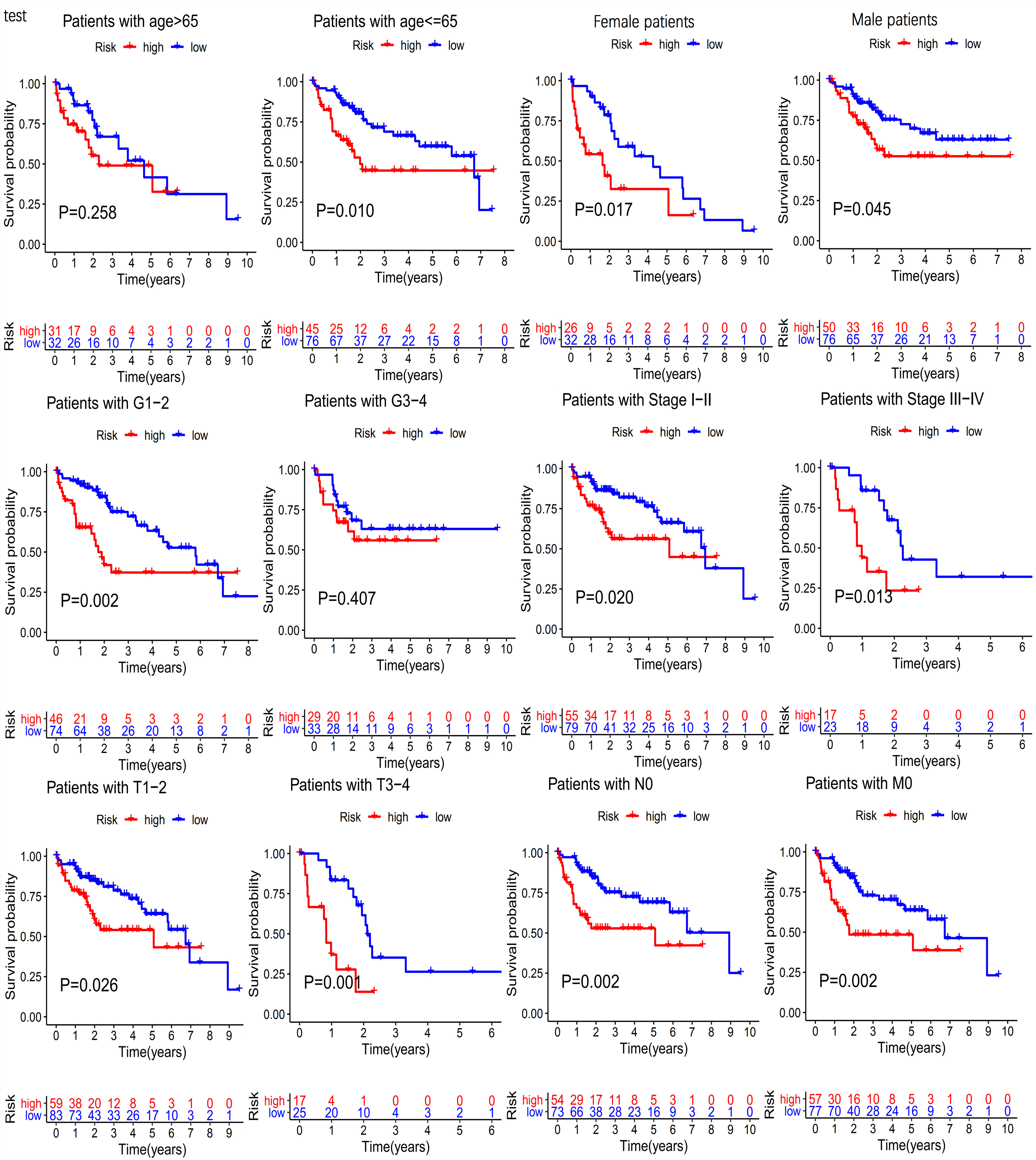

Supplement: Supplementary Figure 3 — Subgroup analysis of the test set. [file Image_3.TIF]

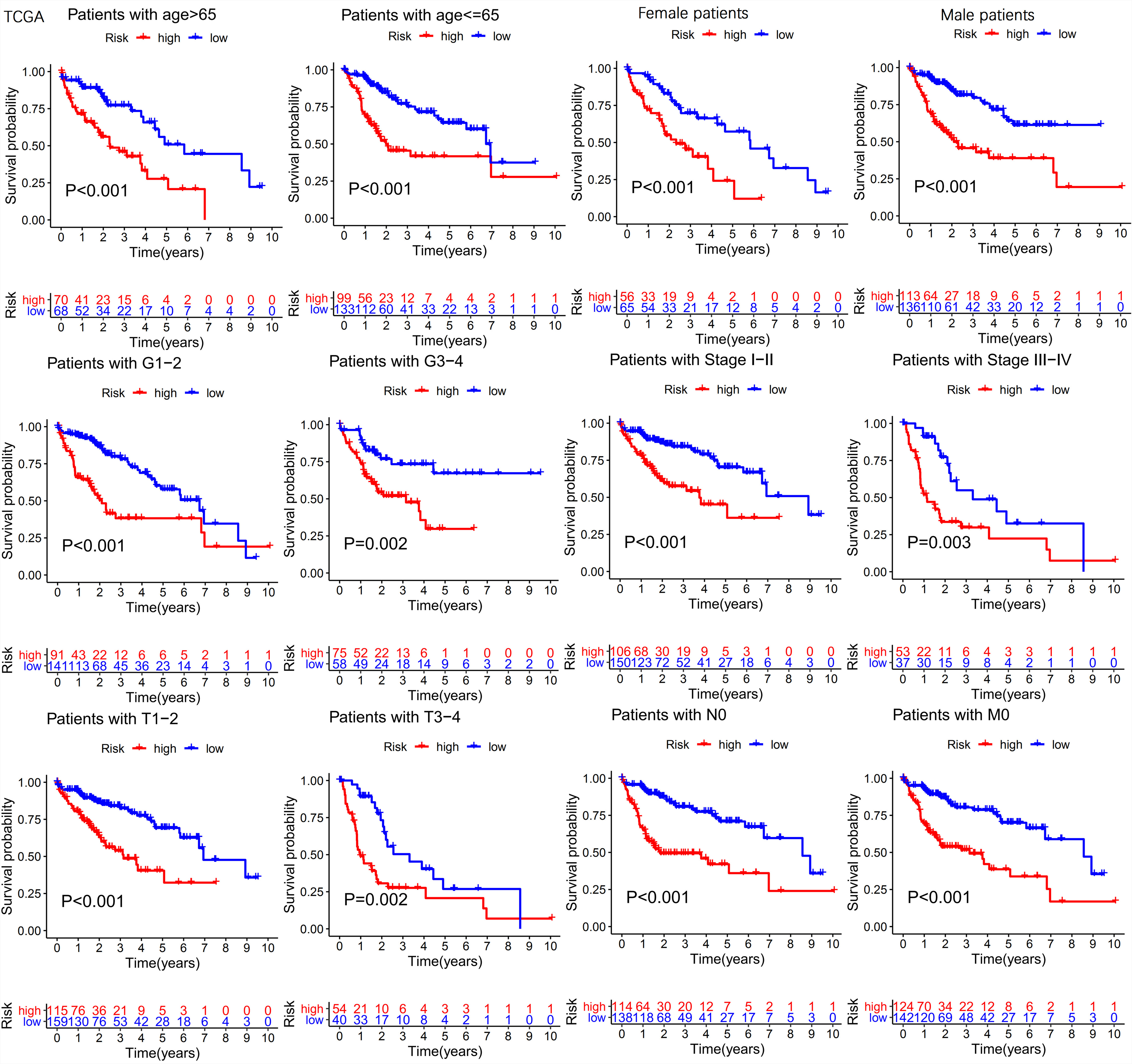

Supplement: Supplementary Figure 4 — Subgroup analysis the TCGA set. [file Image_4.TIF]
