# Supplementary material for: Genomic Instability of Mutation-Derived Gene Prognostic Signatures for Hepatocellular Carcinoma
Source: Front Cell Dev Biol. 2021 Oct 5;9:728574. doi: 10.3389/fcell.2021.728574 (PMC8523793; doi:10.3389/fcell.2021.728574)
Supplement: Supplementary file 5 [file Table_1.docx]

**Supplementary table 1 Primary antibodies of genes in GIGSig**

| Gene | Brand | Source | Concentration |
| --- | --- | --- | --- |
| SLCO2A1 | bioss | rabbit | 1:1000 |
| RPS6KA2  EPHB6 | proteintech  bioss | rabbit  rabbit | 1:1000  1:1000 |
| SLC2A5  PDZD4  CST2  MARVELD1  MAGEA6  SEMA6A | bioss  bioss  proteintech  bioss  proteintech  sinobiologicaL | rabbit  rabbit  rabbit  rabbit  rabbit  rabbit | 1:1000  1:1000  1:1000  1:1000  1:1000  1:1000 |
